# Phytoecdysteroids Accelerate Recovery of Skeletal Muscle Function Following *in vivo* Eccentric Contraction-Induced Injury in Adult and Old Mice

**DOI:** 10.3389/fresc.2021.757789

**Published:** 2021-11-08

**Authors:** Kevin A. Zwetsloot, R. Andrew Shanely, Joshua S. Godwin, Charles F. Hodgman

**Affiliations:** ^1^Integrative Muscle Physiology Laboratory, Appalachian State University, Boone, NC, United States; ^2^Department of Health and Exercise Science, Appalachian State University, Boone, NC, United States; ^3^Department of Biology, Appalachian State University, Boone, NC, United States

**Keywords:** muscle damage, muscle injury, exercise, 20-hydroxyecdysone (20E), muscle aging, muscle regeneration

## Abstract

**Background:** Eccentric muscle contractions are commonly used in exercise regimens, as well as in rehabilitation as a treatment against muscle atrophy and weakness. If repeated multiple times, eccentric contractions may result in skeletal muscle injury and loss of function. Skeletal muscle possesses the remarkable ability to repair and regenerate after an injury or damage; however, this ability is impaired with aging. Phytoecdysteroids are natural plant steroids that possess medicinal, pharmacological, and biological properties, with no adverse side effects in mammals. Previous research has demonstrated that administration of phytoecdysteroids, such as 20-hydroxyecdysone (20E), leads to an increase in protein synthesis signaling and skeletal muscle strength.

**Methods:** To investigate whether 20E enhances skeletal muscle recovery from eccentric contraction-induced damage, adult (7–8 mo) and old (26–27 mo) mice were subjected to injurious eccentric contractions (EC), followed by 20E or placebo (PLA) supplementation for 7 days. Contractile function via torque-frequency relationships (TF) was measured three times in each mouse: pre- and post-EC, as well as after the 7-day recovery period. Mice were anesthetized with isoflurane and then electrically-stimulated isometric contractions were performed to obtain *in vivo* muscle function of the anterior crural muscle group before injury (pre), followed by 150 EC, and then again post-injury (post). Following recovery from anesthesia, mice received either 20E (50 mg•kg^−1^ BW) or PLA by oral gavage. Mice were gavaged daily for 6 days and on day 7, the TF relationship was reassessed (7-day).

**Results:** EC resulted in significant reductions of muscle function post-injury, regardless of age or treatment condition (*p* < 0.001). 20E supplementation completely recovered muscle function after 7 days in both adult and old mice (pre vs. 7-day; *p* > 0.05), while PLA muscle function remained reduced (pre vs. 7-day; *p* < 0.01). In addition, histological markers of muscle damage appear lower in damaged muscle from 20E-treated mice after the 7-day recovery period, compared to PLA.

**Conclusions:** Taken together, these findings demonstrate that 20E fully recovers skeletal muscle function in both adult and old mice just 7 days after eccentric contraction-induced damage. However, the underlying mechanics by which 20E contributes to the accelerated recovery from muscle damage warrant further investigation.

## Introduction

Lengthening (eccentric) muscle contractions elicit higher force production, with lower energy cost, than shortening (concentric) contractions. While eccentric muscle contractions are performed on a daily basis (e.g., descending stairs), eccentric exercise has also been utilized in rehabilitative settings as an effective countermeasure against muscle atrophy and weakness, as well as a modality to treat tendinopathies (1). If repeated multiple times, eccentric contractions are a useful tool to induce a physiologically-relevant injury to skeletal muscle, resulting in damage to the contractile apparatus and loss of function (2, 3). Skeletal muscle possesses the remarkable ability to repair and regenerate after an injury or damage; however, this ability is impaired with aging (4–7). The repair/regeneration of skeletal muscle tissue after damage relies on a series of highly-coordinated and time-dependent processes (8).

While the exact mechanisms responsible for the impaired regenerative response in aged skeletal muscle continue to be explored, it appears that changes to inflammatory processes (9), protein metabolism (10), and endogenous hormones (11) all play significant roles. Evidence suggests that age-related alterations in immune cell (i.e., macrophage) phenotype may contribute to the impaired regenerative capacity of aged skeletal muscle. Greater increases in M2-like macrophages were observed in aged skeletal muscle, compared to young, in both humans (12) and mice (13) at the same time point during regeneration following eccentric damage. Interestingly, no age-related differences in pro-inflammatory cytokine expression (IL-1B, TNFα, IFNγ) have been observed in regenerating mouse muscle tissue (14). Additionally, the healthy maintenance of skeletal muscle mass is achieved by an intricate balance between protein synthesis and protein breakdown. It has been demonstrated extensively that aged skeletal muscle displays a reduced ability for anabolic stimuli [e.g., resistance exercise (15, 16) and dietary protein ingestion (17, 18)] to stimulate protein synthesis, resulting in a negative protein balance; a phenomenon aptly named “anabolic resistance.” Lastly, correlations exist between declining gonadal hormones and diminished skeletal muscle health in elderly men (11) and women (19). Some previous studies have reported efficacy of hormone therapy (HT) on improving or maintaining skeletal muscle health in certain aged populations (20–23); however, the long-term potential for adverse side effects with HT [e.g., increased risk for the development of certain types of cancers and cardiovascular events (24, 25)] may outweigh the possible benefits. Clearly, the underlying mechanisms responsible for impaired regeneration in aged skeletal muscle are multifaceted. While treatments that target exercise, dietary protein intake, and endogenous hormones have shown marginal success, there is still a great need for developing effective, natural interventions to enhance muscle regeneration with aging.

Phytoecdysteroids (PEs) are natural plant steroids found in a variety of hardy plant species, such as *Ajuga* and *Leuzea*, as well as commonly consumed spinach (*Spinacia oleracea*). PEs possess a plethora of medicinal, pharmacological, and biological properties, with no adverse side effects in mammals (26, 27). Characterized as polyhydroxylated basic carbon ring structures of 27-29 carbons, PEs elicit immunoprotective, antioxidant, anabolic, hepatoprotective, hypoglycemic, and physical performance enhancing effects (28). While over 250 different PEs have been identified, 20-hydroxyecdysone (20E) is the most widely investigated. It has been suggested that the anabolic effects of 20E are mediated via a G-protein coupled cell surface receptor (29), as opposed to an intracellular androgen receptor. Thus, 20E is considered anabolic, but non-androgenic since it does not increase prostate or seminal vesicle mass in young castrated rats after 10 days of treatment (30), nor does it alter organ or testes mass in aging mice with 28 days of treatment (31). Regarding skeletal muscle, 20E has been reported to increase grip strength in young rats and stimulate protein synthesis via the PI3K/Akt pathway in C2C12 myotubes *in vitro* (32). Further, Toth et al. (33) reported that 7 days of 20E treatment increases fiber cross sectional area in healthy soleus, but not the extensor digitorum longus muscle, as well as enhances muscle growth in regenerating (myotoxin-injected) soleus muscles of young rats. Conversely, we recently reported that 28 days of 20E treatment does not alter muscle mass or fiber size, nor does a single acute treatment of 20E stimulate anabolic signaling in skeletal muscle tissue from sedentary aging C57BL/6 mice (31). From these findings, we concluded that a concurrent stress (e.g., recovery from damage) may be required for 20E to elicit beneficial effects on skeletal muscle.

Taken together, it appears that 20E may have the potential to modulate muscle regeneration. With a few exceptions [e.g., (33)], most studies to date have only examined the effect of phytoecdysteroids on muscle size or mass in sedentary, healthy skeletal muscle. No studies have assessed the functional characteristics of skeletal muscle after injury/damage with phytoecdysteroid treatment. Therefore, the purpose of this study was to investigate if 20E accelerates the functional recovery of skeletal muscle after eccentric damage in adult and old mice.

## Materials and Methods

### Experimental Design

Male C57BL/6 adult [7.4 ± 0.1 and 7.8 ± 0.4 months of age in the PLA (*n* = 4) and 20E (*n* = 7) treatment groups, respectively] and old [26.4 ± 0.4 and 26.5 ± 0.4 months of age in the PLA (*n* = 7) and 20E (*n* = 7) treatment groups, respectively] were used in this study. The ages of the adult mice in this study were the equivalent of 30–35 year-old humans, while the old mice were the equivalent of 70–75 year-old humans (9). First, mice were anesthetized (4% isoflurane and maintained with 2% isoflurane) and pre-eccentric damage *in vivo* contractile function of the anterior crural muscle group [tibialis anterior (TA), extensor digitorum longus (EDL), and extensor hallucis longus] was measured. Mice were immediately subjected to the eccentric contraction-induced muscle damage protocol, and then *in vivo* contractile function was reassessed. Upon completion of the post-eccentric damage contractile function test, mice were allowed to recover from anesthesia and then returned to their cage. Once fully recovered from anesthesia, mice were randomly assigned to either the placebo control treatment group (PLA) or the 20-hydroxyecdysone treatment group (20E) and received the first treatment via oral gavage. Mice were administered daily treatment doses, at approximately the same time of day, for 6 days. Twenty-four hours after the seventh and final treatment dose, mice were anesthetized again and 7-day recovery *in vivo* contractile function was measured, as described above (repeated measures design). Finally, mice were sacrificed under anesthesia and the TA and EDL muscles were harvested, weighed for mass, and mounted for histological analysis.

### Phytoecdysteroid Treatment

Mice assigned to the 20E treatment groups received daily doses of 50 mg • kg^−1^ body mass (BM) 20E (E6425-HE; Bosche Scientific, New Brunswick, NJ, USA) dissolved in phosphate-buffered saline (PBS) via oral gavage for 7 days. Mice assigned to the vehicle control treatment groups (placebo; PLA) received daily doses of equivalent volume PBS for 7 days. BM was recorded each day. The dose of 50 mg • kg^−1^ BM is based on the previous studies by Gorelick-Feldman et al. (32) and Lawrence et al. (31).

### *In vivo* Contractile Function Testing and Eccentric Contraction-Induced Damage Protocol

Contractile function of the anterior crural muscles was measured three times in each mouse: pre- and post-eccentric damage, as well as after the 7-day treatment period, via the torque-frequency relationship, as previously described (34, 35). Under anesthesia, hair on the left hindlimb was removed with dilapidation cream and thoroughly rinsed with water. Mice were then placed on a heated (37°C) platform and the left foot was secured with an aluminum foot-cover and tape to the footplate affixed to the shaft of a dual-mode servomotor (300B-LR, Aurora Scientific, Aurora, ON, Canada). A clamp secured to a micro-manipulator (World Precision Instruments, Sarasota, FL) was used to position and hold the left knee in place during the procedure. The ankle joint was held at 90° of passive dorsiflexion with respect to the tibia and the tibia was positioned at 90° with respect to the femur. Sterilized 30-gauge needle electrodes (Grass Instruments, Warwick, RI) were inserted through the skin for stimulation of the left common peroneal nerve, each was positioned and held in place with a micro-manipulator. Single isometric contractions (1 Hz) were used to obtain initial needle electrode placement; optimal stimulation voltage (5–10 volts) and needle electrode placement was confirmed by 5–10 isometric contractions (200-ms train duration, 0.1-ms pulse width at 300 Hz). Following needle electrode placement, a torque-frequency curve measured peak isometric torque produced by the anterior crural muscle group at 10 ascending stimulation frequencies, from 20 to 300 Hz, with 2 min rest between each contraction.

**Figure 1:**
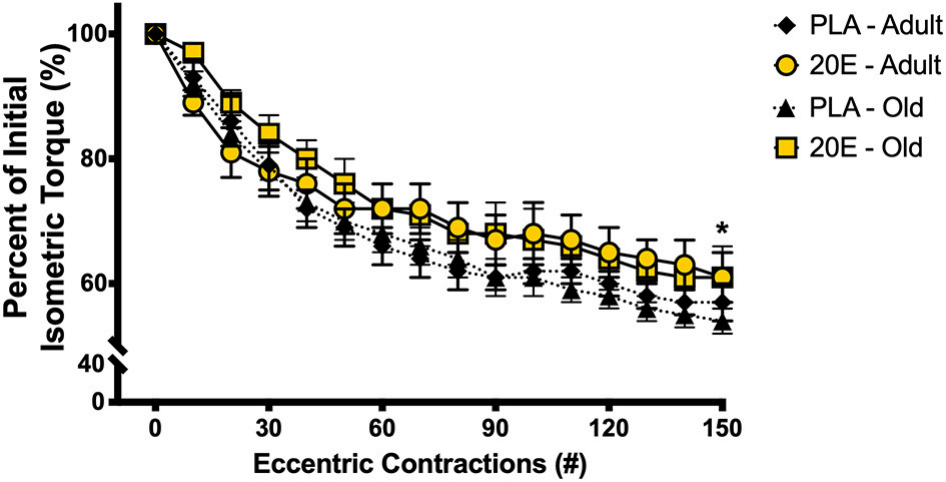
Percent of initial isometric torque during the 150 eccentric contraction-induced muscle damage protocol. Maximal tetanic isometric torque of the anterior crural muscles was measured after every 10th eccentric contraction of the damage protocol in adult and old mice. There was a significant 40–50% decline in isometric torque after the 150-eccentric contraction muscle damage protocol, regardless of age or treatment condition. Values represent mean ± SEM. PLA, placebo; 20E, 20-hydroxyecdysone. * significantly different than initial; *p* < 0.001.

After completion of the initial pre-eccentric damage *in vivo* contractile function testing, the anterior crural muscle group was immediately subjected to the eccentric contraction-induced muscle damage protocol using 150 eccentric contractions (300 Hz, 120-ms train of 0.1-ms pulses, with 38° of angular movement at 2,000°•s^−1^ starting in 19° of dorsiflexion, moving to 19° of plantarflexion) described by Corona et al. (35). Muscle function was also assessed every 10th eccentric contraction *via* individual 300 Hz isometric contractions (Figure 1). Warren et al. previously reported that decreases in isometric torque during this eccentric contraction-induced muscle damage protocol are the result of muscle injury, not fatigue (36). After completion of the eccentric contraction-induced damage protocol and a 5-min delay, post-eccentric damage *in vivo* contractile function was measured. Finally, after the 7-day treatment period, mice were anesthetized and 7-day recovery *in vivo* contractile function was measured as described above.

Additionally, separate groups (*n* = 10/each) of sham-treated adult and old mice were utilized as experimental controls. Mice in the sham-treated groups performed all of the same experimental procedures described above (including the 7-day 20E and PLA treatments), except they were not subjected to the eccentric damage protocol. During the time required to complete the eccentric damage protocol (~30 min), mice remained anesthetized and resting before continuing with the post- and eventually the 7-day sham-recovery contractile function tests.

### Contractile Function Data Acquisition and Analysis

**Figure 2:**
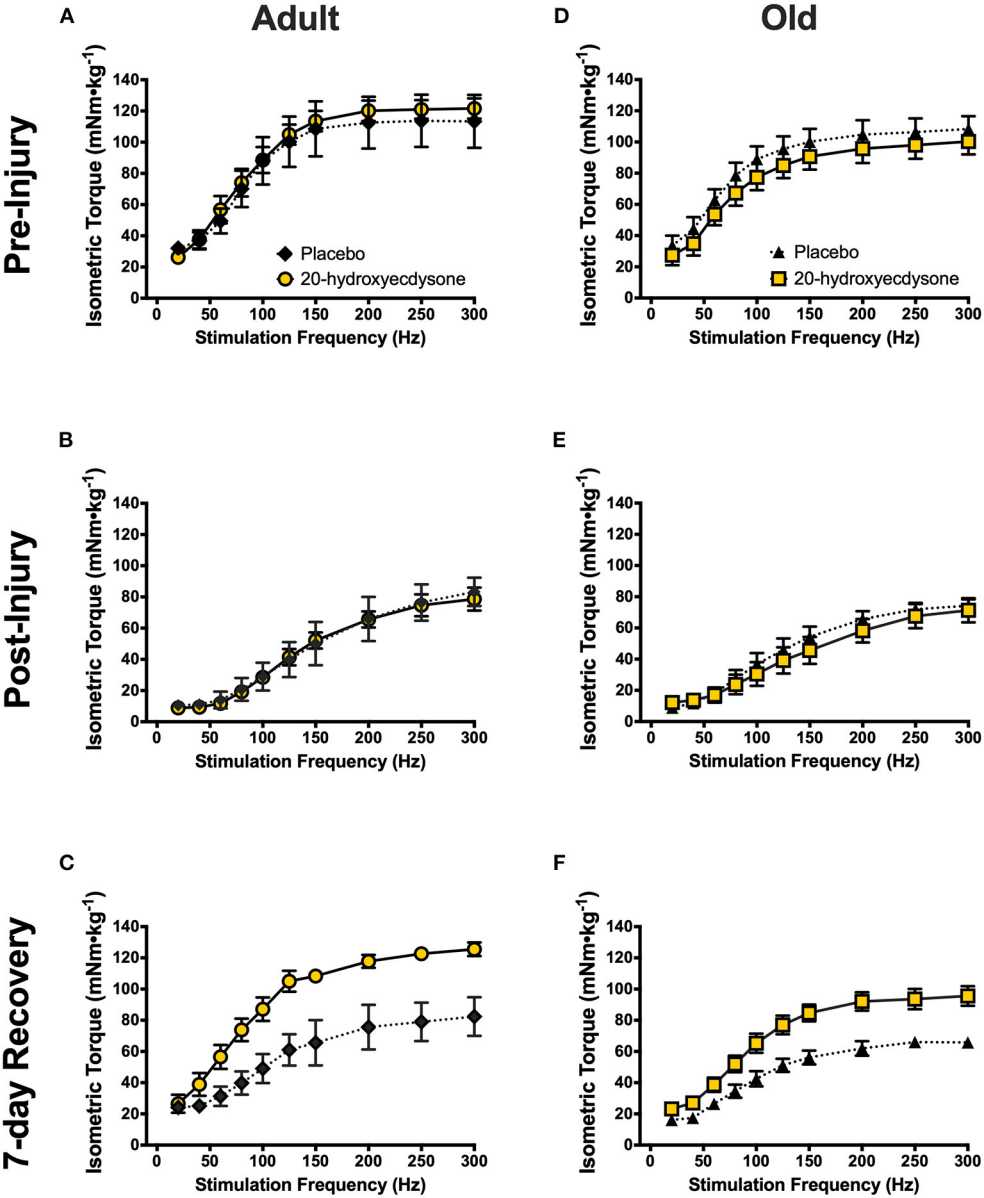
Isometric torque-frequency relationships of the anterior crural muscles in adult and old mice at the pre-injury (**A,D**, respectively), immediately post-injury (**B,E**, respectively), and 7-day recovery (**C,F**, respectively) time points. Analysis revealed significant reductions in isometric torque-frequency relationships in both adult **(B)** and old mice **(E)** immediately after the eccentric contraction-induced muscle damage protocol (Post-Injury), compared to Pre-Injury (**A,D**, respectively; *p* < 0.001), regardless of treatment condition. Torque-frequency relationships fully recovered to Pre-Injury levels by the 7-day Recovery time point in both 20E-treated adult **(C)** and old mice **(F)**, but PLA-treated mice did not recover (*p* = 0.005). Values represent mean ± SEM. PLA, placebo; 20E, 20-hydroxyecdysone.

The muscle lever system (Aurora Scientific 1300A), stimulator and force transducer were connected to a signal interface (Aurora Scientific, Model 610A) that sends the analog signal to an analog-to-digital converter card (National Instruments, Austin, TX) on a computer with Dynamic Muscle Control software (Aurora Scientific, DMC 610A). The force output data were analyzed utilizing Dynamic Muscle Analysis software (Aurora Scientific, DMA 610A). Raw (group mean) torque-frequency relationships displayed in Figure 2 and Supplementary Figure 1 were analyzed for statistical significance prior to modeling. To generate the EC_50_, torque-frequency relationship data were modeled with the following four-parameter logistic fit equation using GraphPad Prism version 8.4.3, GraphPad Software, San Diego, California USA:

$$\begin{array}{l} f\left(\text{x}\right)\;=\;\min\;+\;\left(\max\;-\;\min\right)/\left[1\;+\;\left(\text{x}/\text{EC}50\right)n\right] \end{array}$$

where x is the stimulation frequency; min and max are the smallest (i.e., twitch) and largest (i.e., peak tetanic) torques estimated from the best-fit torque-frequency relationship curve, respectively; EC_50_ is the stimulation frequency required to generate 50% of maximal estimated torque (max–min); and *n* is the Hill Slope Coefficient indicating the slope of the steepest portion in the estimated torque-frequency relationship curve (37). The EC_50_ provides further insight on contractile function from the estimated torque-frequency relationships (38).

### Histological Analyses

The TA muscle was harvested from the left hindlimb and mounted for histological analysis following measurement of 7-day recovery *in vivo* contractile function. Harvested tissues were mounted on cork using a mixture of tragacanth gum and optimal cutting temperature medium (Fisher Scientific, Houston, TX), frozen in liquid nitrogen-cooled isopentane, and stored at −80°C until sectioning. TA muscle samples were cut into 10 μm cross-sections using a cryostat (CryoStar Model HM 505; ThermoFisher Scientific Inc.) and mounted on positively charged microscope slides.

Muscle section quality, tissue integrity, and markers of muscle damage were assessed using common histological techniques for cytosolic and nuclear components using Mayer's hematoxylin and eosin (H&E) solutions (Millipore Sigma, St. Louis, MO). H&E-stained muscle sections were imaged with 4 × and 10 × objectives using an Olympus IX81 light microscope and cellSens Imaging Software (Olympus, Waltham, MA). Markers of muscle damage (e.g., edema, overt fiber damage, presence of infiltrating inflammatory cells, and centrally-located myonuclei) (39–41) were assessed in each image using qualitative indices on a scale of 0–3 to provide a muscle damage score: with 0 = no apparent muscle damage; 1 = minimal muscle damage; 2 = moderate muscle damage; and 3 = severe muscle damage.

Skeletal muscle repair and regeneration after damage is often accompanied by remodeling of connective tissue. Gomori trichrome staining (Millipore Sigma, St. Louis, MO) was performed on TA muscle sections (10 μm) as this technique differentiates skeletal muscle from connective tissue. Gomori trichrome-stained muscle sections were imaged with 4 × and 10 × objectives using an Olympus IX81 light microscope and cellSens Imaging Software. No analyses were performed on Gomori trichrome-stained sections.

### Statistical Analyses

All data are expressed as mean ± SEM. Torque-frequency relationship data were analyzed using a repeated measures three-way factorial ANOVA (treatment, two levels; time, three levels; stimulation frequency, 10 levels). Measures of contractile performance data (eccentric damage isometric contractions, twitch torque, maximal tetanic torque, twitch:tetanic ratio, and EC_50_) and animal body masses were analyzed using a repeated measures three-way factorial ANOVA (treatment, two levels; time, two or three levels; age, two levels). Individual muscle masses were analyzed using a repeated measures two-way factorial ANOVA (treatment, two levels; age, two levels). The *a priori* level of significance was set at *p* < 0.05. Following a significant F-ratio, Fisher's LSD pairwise *post-hoc* comparisons were made. Data were analyzed using SPSS (IBM Corp., Armonk, NY). No statistical analyses were performed for the qualitative analysis for muscle damage scores.

## Results

### Animal Subjects and Skeletal Muscle Mass

Animal subject characteristics are shown in Table 1. Body mass did not differ between any treatment group (time x treatment x age interaction, *p* = 0.875); however, all groups lost body mass between initial and the 7-day recovery time point (main effect of time, *p* = 0.008). Furthermore, TA and EDL muscle mass (normalized to body mass; mg•g^−1^) were significantly lower in the old mice (main effect of age, *p* = 0.003 for both TA and EDL), regardless of treatment (treatment x age interaction, *p* = 0.453 for TA and *p* = 0.326 for EDL). Animal and muscle characteristics for sham-treated groups followed similar patterns of significance as the eccentric damage groups stated above (Supplementary Table 1).

**Table 1:** Animal and muscle characteristics of eccentric damage groups.

	**Adult**		**Old**	
	**PLA (*n* = 4)**	**20E (*n* = 7)**	**PLA (*n* = 7)**	**20E (*n* = 7)**
Age (mo)	7.4 ± 0.1	7.8 ± 0.4	26.4 ± 0.4	26.5 ± 0.4
Initial body mass (g)	30.1 ± 1.3	28.5 ± 0.9	30.0 ± 0.8	30.8 ± 1.2
7-day body mass (g)	29.6 ± 1.1^$^	27.5 ± 0.8^$^	29.4 ± 0.7^$^	29.6 ± 0.8^$^
TA muscle mass (mg•g^−1^ body mass)	1.69 ± 0.07	1.64 ± 0.05	1.39 ± 0.10^#^	1.45 ± 0.05^#^
EDL muscle mass (mg•g^−1^ body mass)	0.36 ± 0.03	0.39 ± 0.03	0.30 ± 0.02^#^	0.28 ± 0.01^#^

*Values represent mean ± SEM. PLA, placebo; 20E, 20-hydroxyecdysone; TA, tibialis anterior; EDL, extensor digitorum longus*.

$ *Significantly different than initial (p < 0.01)*.

# *Significantly different than adult (p < 0.01 for TA and EDL)*.

### Eccentric Contraction-Induced Muscle Damage

The eccentric contraction-induced muscle damage protocol results in a ~40–50% decline (main effect of time, *p* < 0.001) in anterior crural muscle group contractile function, measured by maximal isometric torque (Figure 1). Additionally, there were no differences in the loss of contractile function in response to eccentric damage between ages of mice or treatment groups (time x treatment x age interaction, *p* = 0.740).

### *In vivo* Skeletal Muscle Contractile Function

To investigate if 20E accelerates the recovery of skeletal muscle after eccentric damage in adult and old mice, *in vivo* skeletal muscle contractile function was measured before, immediately after, and at 7 days of recovery from eccentric damage. Indeed, the eccentric contraction-induced damage protocol significantly reduced isometric torque immediately post-damage in both adult and old mice (main effect of time, *p* < 0.001; Figures 2B,E, respectively; time × treatment × age interaction, *p* = 0.753). Most remarkable, however, was the significant time × treatment interaction (*p* < 0.001) demonstrating that 20E treatment resulted in full recovery of isometric torque at 7 days-post eccentric damage in both adult and old mice, while neither PLA-treated group (both adult and old) recovered by 7 days (Figures 2C,F, respectively). Additionally, we performed experiments on adult and old sham-treated mice that had not performed the eccentric damage protocol. *In vivo* skeletal muscle contractile function was not altered over time or treatment in any of the adult or old sham-treated groups (time × treatment × age interaction, *p* = 0.766), thus demonstrating the repeatability of our experimental procedures (Supplementary Figure 1).

**Figure 3:**
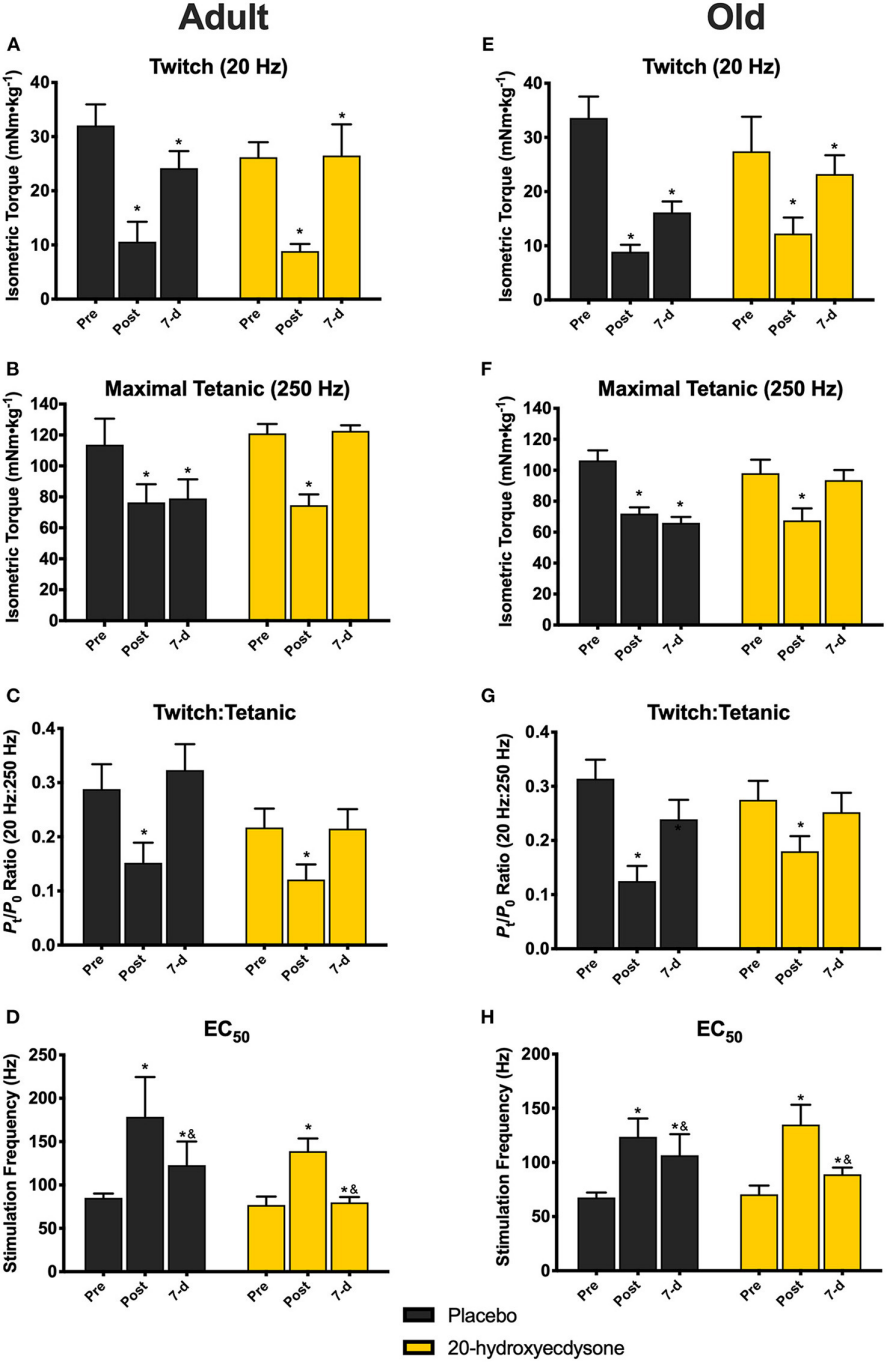
Isometric twitch torque, maximal tetanic torque, twitch:tetanic ratio, and EC_50_ of the anterior crural muscles in Adult and Old mice at the pre-injury, immediately post-injury, and 7-day recovery time points. Analysis revealed significant reductions in twitch torque (20 Hz; **A,E**) in all groups immediately after (Post) and at 7 days (7-d) following the eccentric contraction-induced muscle damage protocol, regardless of treatment condition. Maximal tetanic torque (250 Hz) was also significantly reduced in both adult **(B)** and old mice **(F)** Post eccentric damage, compared to Pre, regardless of treatment condition. Only the 20E-treated adult and old mice fully recovered maximal tetanic torque to pre-injury levels by 7-d. Twitch:Tetanic ratio was significantly reduced at Post in both adult **(C)** and old mice **(G)**, regardless of treatment condition, compared to Pre, but Twitch:Tetanic recovered by 7-d. EC_50_ was significantly higher in all age and treatment groups at Post, but EC_50_ at 7-d was still significantly different than Post and Pre **(D,H)**. Values represent mean ± SEM. PLA, placebo; 20E, 20-hydroxyecdysone; EC_50_, stimulation frequency required to elicit 50% of maximal tetanic torque. * significantly different than Pre within the same group (*p* < 0.05); & significantly different than Post within the same group (*p* < 0.05).

Further analysis of contractile function revealed that there was only a significant main effect of time (*p* < 0.001) in twitch torque (20 Hz), as twitch was significantly lower in all treatment groups and ages immediately after eccentric damage (*p* < 0.001) and remained lower at 7 days (*p* = 0.032; Figures 3A,E, respectively), compared to pre-damage. While no time x treatment x age interaction (*p* = 0.677) existed in maximal tetanic torque (250 Hz), there was a significant time x treatment interaction (*p* < 0.001). Maximal tetanic torque was significantly lower immediately after eccentric damage in all treatment groups and ages (*p* < 0.001); however, only the 20E-treated groups in both adult (Figure 3B) and old mice (Figure 3F) fully recovered maximal tetanic torque by returning to pre-damage levels at 7 days of recovery. Similar to twitch torque, there was only a significant main effect of time in the twitch to tetanic ratio (*p* < 0.001), as the ratio was significantly lower in all groups immediately after eccentric damage (Figures 3C,G), but recovered with 7 days of recovery (time × treatment × age interaction, *p* = 0.630). Furthermore, there were no age- or treatment-related differences regarding how eccentric damage caused muscle contractile dysfunction or recovery from muscle damage (all treatment x age interactions, *p* > 0.500). In other words, adult and old mice displayed similar responses to eccentric muscle damage and recovery with 20E treatment.

There was no time x treatment x age interaction (*p* = 0.586) for EC_50_; however, there was a significant main effect of time (*p* < 0.001), such that EC_50_ was significantly higher in all age and treatment groups immediately post-damage (*p* < 0.001), before beginning to return back to pre-damage levels by 7 days of recovery, i.e., there was still a significant difference between 7-d vs. Pre (*p* = 0.009), but also a difference between 7-d vs. Post (*p* = 0.002) time points (Figures 3D,H). These data signify that a greater stimulation frequency is required to elicit 50% of maximal tetanic torque immediately after eccentric damage.

### Histology

**Figure 4:**
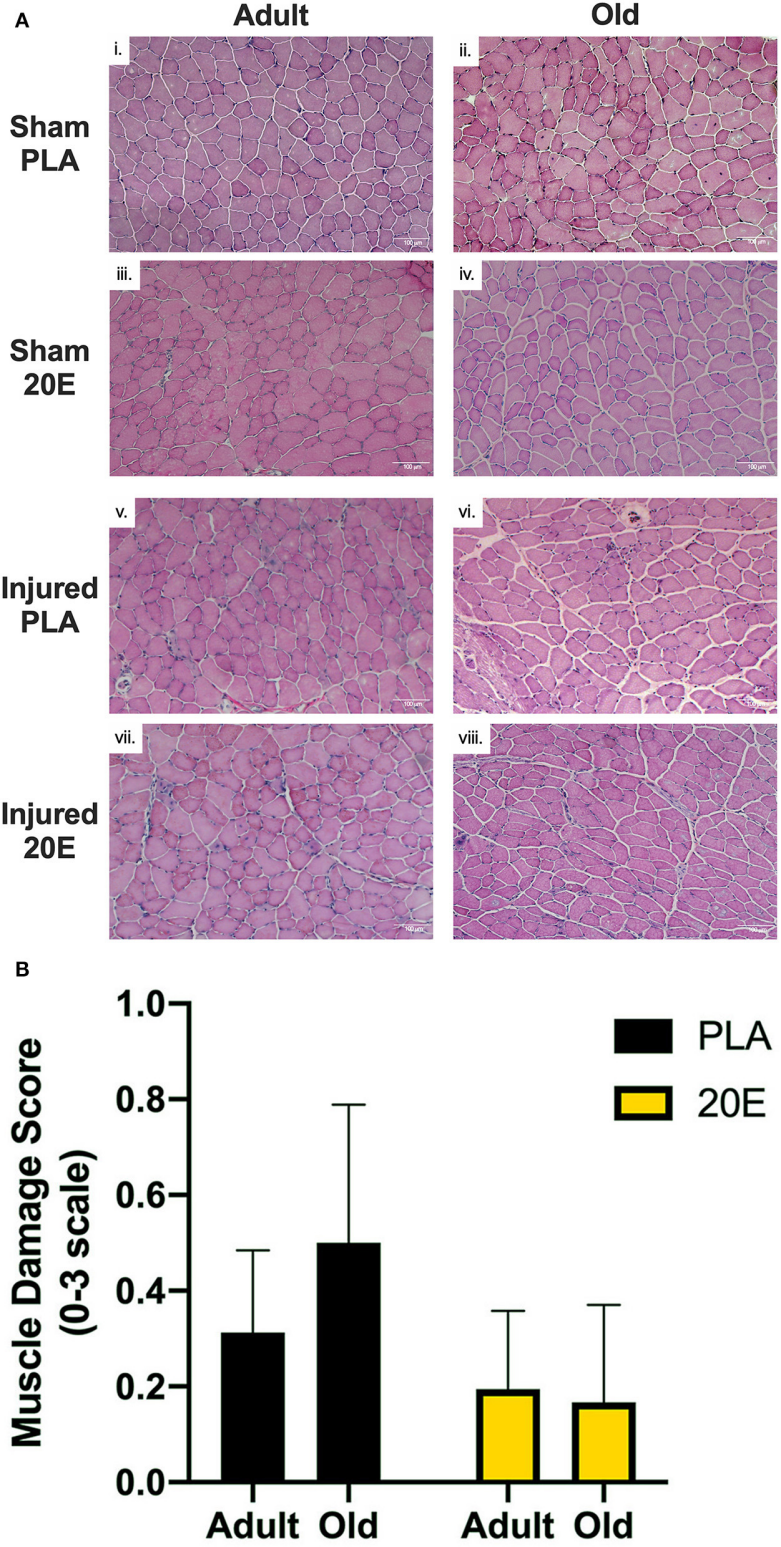
Representative images for H&E-stained tibialis anterior (TA) muscle sections from sham-treated (Sham; i–iv) and eccentric damaged (Injured; v–viii) groups after the 7-day recovery period **(A)** and muscle damage scores of injured TA muscle sections **(B)**. H&E staining procedures provide visualization of the nuclei (dark blue) and cytosol (pink). **(A)** Injured TA muscle sections appear to have more markers of muscle damage (e.g., edema, overt fiber damage, presence of infiltrating inflammatory cells, and centrally-located myonuclei), regardless of treatment, compared to Sham muscle sections. **(B)** Qualitative analysis of markers of muscle damage were assessed in eccentric contraction-injured TA muscle sections to obtain a Muscle Damage Score based on a scale of 0–3, with 0 = no apparent muscle damage; 1 = minimal muscle damage; 2 = moderate muscle damage; and 3 = severe muscle damage. Muscle damage scores appear to be higher in the PLA-treated groups, compared to 20E-treated groups, but the old PLA-treated mice appear to have the greatest muscle damage score at 7 days post-injury, compared to any other group. Values represent mean ± SEM. PLA, placebo; 20E, 20-hydroxyecdysone. Scale bar = 100 μm.

H&E staining was performed to observe morphological muscle damage (i.e., edema, fiber damage, presence of infiltrating inflammatory cells, and centrally-located myonuclei). Markers of muscle damage were minimally observed (muscle damage scores ≤ 1.0 on a scale of 0–3) at 7 days post-injury in TA muscles subjected to eccentric damage (Figures 4Av–viii), compared to sham-treated muscles (Figures 4Ai–iv). Muscle damage scores obtained via qualitative/semi-quantitative analyses revealed that muscles in the PLA-treated groups appear to have higher muscle damage scores, compared to 20E-treated groups (39–41). Furthermore, the old PLA-treated mice appeared to have the greatest muscle damage score at 7 days post-injury, compared to any other group (Figure 4B).

**Figure 5:**
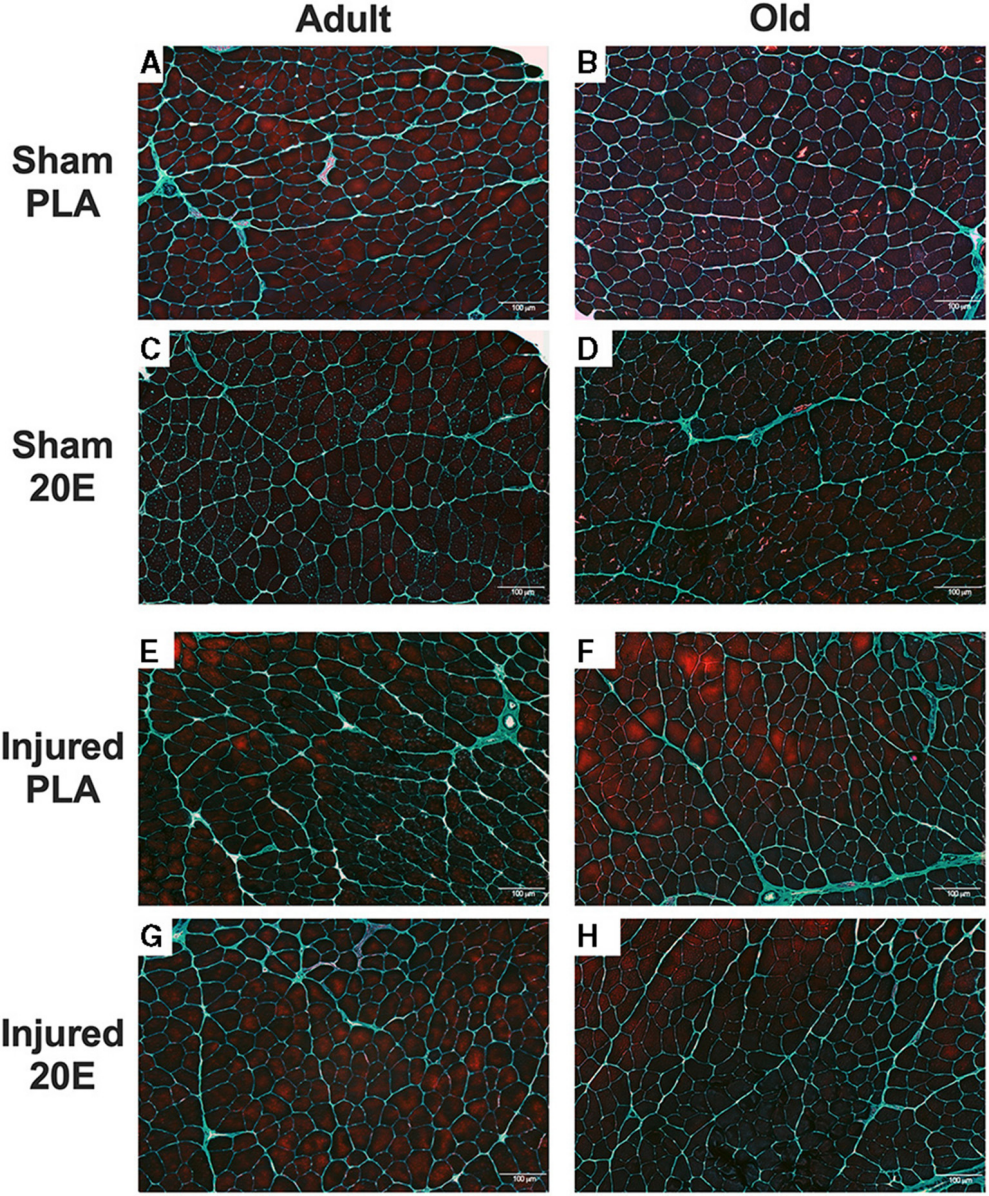
Representative images for Gomori trichrome-stained tibialis anterior (TA) muscle sections from sham-treated (Sham; **A–D**) and eccentric damaged (Injured; **E–H**) groups after the 7-day recovery period. Gomori trichrome staining procedures provide visualization of the nuclei (dark blue), muscle fibers (reddish purple), and the connective tissue (greenish blue). Injured TA muscle sections appear to have connective tissue staining regardless of treatment, compared to Sham muscle sections. PLA, placebo; 20E, 20-hydroxyecdysone. Scale bar = 100 μm.

Gomori trichrome staining of the TA muscles revealed that connective tissue staining appears to be higher in the muscles subjected to eccentric damage (Figures 5E–H)„ compared to sham-treated muscles (Figures 5A–D). It does not appear that 20E treatment has any effect on connective tissue staining in regenerating muscle tissue at 7 days post-injury.

## Discussion

When skeletal muscle is subjected to contractile stress and/or strain that supersedes the normal capabilities of the muscle, an injury or damage occurs and functional capacity (i.e., muscle torque/force or strength) is diminished. Here in this study, as with many others (34, 35, 42–44), we reaffirm that functional capacity is significantly impaired immediately, and for several days after, 150 eccentric contractions. This eccentric contraction-induced damage protocol results in ~40–50% reductions in isometric torque of the anterior crural muscles (Figure 1). Consistent with previous studies in rodents (45, 46), these declines in functional capacity with eccentric contractions were similar between the adult and old mice and between treatments. In other words, all mice in this study sustained similar degrees of muscle damage, regardless of age or treatment. It is important to note that 20E (or placebo) treatments did not begin until the mouse had recovered from anesthesia after the initial muscle function testing session, which included the eccentric damage protocol. Therefore, it is not possible for 20E to have provided any prophylactic or protective effects on muscles suffering eccentric damage. Arguably, functional loss of strength is the most important and reliable indicator of skeletal muscle injury, not to mention the clinical implications that loss of muscle function imparts (3). The force-producing capabilities of the muscle usually return to baseline (pre-injury) levels between 2 and 4 weeks post-eccentric contraction-induced injury (34, 42, 44, 46, 47). But this process may take longer depending on the severity of damage incurred (48). The regenerative capacity of skeletal muscle is impaired during aging, and this can lead to prolonged or incomplete recovery from eccentric damage, as well as further loss of muscle mass and strength. Seminal work by Brooks and Faulkner (46) demonstrated that while young and aged mice respond similarly to eccentric damage (i.e., loss of strength and markers of muscle damage), young mice had recovered muscle function by 4 weeks post-damage, whereas aged mice had not. The most intriguing and novel finding of the current study was that 20-hydroxyecdysone (20E) completely recovered skeletal muscle functional capacity by 7 days post-injury, not only in the adult mice, but also in the old mice. Based on only measuring a single time point and the relatively short recovery period examined in the current study (7 days), it is difficult to forecast just how long it would have taken for the placebo-treated mice in our study to recover muscle function back to pre-injury levels. It seems plausible that some, but not full recovery of muscle function is possible in just 7 days after eccentric muscle damage (35, 49, 50). This study demonstrates, for the first time, that oral supplementation with phytoecdysteroids accelerates the functional recovery of skeletal muscle in both adult and old mice after eccentric contraction-induced injury.

A major tenet of muscle contraction and functional capacity is the ability for a neural impulse to stimulate the release of calcium (Ca^+2^) from the sarcoplasmic reticulum (SR) leading to the development of force at the sarcomere, a process widely known as excitation-contraction (EC) coupling. Traditionally, it was thought that disruption of the force-generating and force-transferring structures of the sarcomere was responsible for the loss of muscle function after eccentric contraction-induced muscle damage (51). However, there is a dissociation between muscle function and histological markers of muscle damage that disputes the latter causal relationship. Recall that the greatest declines in muscle function occur immediately following eccentric contraction-induced injury, yet markers of muscle damage are not fully apparent until 1–2 days after injury (34, 47, 52). Studies from the past few decades have established that the early functional loss of strength from eccentric contractions (0–5 days post-injury) is primarily the result of EC coupling dysfunction, not contractile structure disruption. Warren et al. (53) were the first to describe eccentric contraction-induced EC coupling dysfunction in mouse soleus muscle *in vitro*. Later, Ingalls et al. (47), exhibited that the site for EC dysfunction in response to eccentric damage lies at the level of the t-tubule and the SR Ca^+2^ release channel (i.e., the “triad”), specifically via disruption of the interface between dihydropyridine receptors (DHPR) and ryanodine receptors (RyR1). The decreased twitch to tetanic ratio observed immediately after injury provides an indirect indication of EC coupling dysfunction with eccentric damage; however, this impairment was recovered by 7 days, regardless of treatment or age (50, 54). While 20E elicits a rapid, but transient (30–180 s) increase in intracellular [Ca^+2^] in C2C12 myotubes *in vitro* (29), it is highly unlikely that 20E is producing any long-term effects on Ca^+2^ kinetics or repair of the dysfunctional EC coupling mechanism resulting in the observed rescue of muscle function at 7 days in this study.

It has been estimated that EC coupling dysfunction is responsible for 57–75% of the functional loss of muscle strength acutely after eccentric contractions (51). However, in addition to reversing EC coupling dysfunction, other mechanisms also contribute to the recovery of muscle function observed in the days to weeks following eccentric damage. As the normal progression of damage and regeneration processes occur, disruptions to sarcomeric (contractile apparatus) and sarcolemmal (membrane) structures have been observed within days of the eccentric exercise bout (35, 55, 56). Furthermore, there is sufficient evidence that alterations in skeletal muscle protein metabolism may be responsible for the remaining strength deficits in the 1–2 weeks after eccentric damage (34, 42). Therefore, a need to restore protein balance may account for the prolonged recovery time. Skeletal muscle protein synthesis signaling, via activation of the mTORC1 pathway, is stimulated in the range of 1–7 days post-damage in response to various eccentric or lengthening contraction protocols in rodents and humans (57–61). Phytoecdysteroids, particularly 20E, promote anabolic responses in many tissues, including skeletal muscle. Gorelick-Feldman et al. demonstrated that 20E stimulates protein synthesis (32), primarily through the activation of PI3K/Akt signaling (29), in a dose-dependent manner in C2C12 myotubes *in vitro*. Furthermore, pre-treatment of myotubes with the G protein inhibitor, Bordetella pertussis toxin (PTX), abolishes the 20E effect on Ca^+2^ influx and Akt activation, suggesting that 20E functions through a G protein-coupled cell surface receptor (29). We recently reported that 20E does not provide an anabolic stimulus, via activation of the PI3K/Akt/mTORC1 pathway, in skeletal muscle from aging *sedentary* mice, despite using the same dose of 20E as in the current study (50 mg•kg^−1^ BM) (31). From this study, we concluded that an additional stimulus, for example exercise or injury, may be required for 20E to elicit anabolic effects on skeletal muscle. The novel observation that 20E treatment fully recovers skeletal muscle function in just 7 days after eccentric damage is much earlier than other studies using similar eccentric damage protocols in mice (34, 35, 42, 47). Therefore, we conclude that 20E accelerates the functional recovery of muscle after eccentric damage. While we cannot definitively state that the recovery of eccentric contraction-induced skeletal muscle dysfunction after just 7 days with 20E supplementation is due solely to the anabolic effects of 20E (*via* PI3K/Akt/mTORC1 signaling), based on previous literature it seems plausible that 20E may provide an anabolic stimulus to damaged muscle leading to accelerated recovery of muscle function (62). Moreover, since aged skeletal muscle displays anabolic resistance, 20E may be able to stimulate alternative anabolic pathways to those traditionally responsible for the anabolic resistance in aged muscle. Thus, daily 20E treatments, starting immediately after eccentric damage, could be providing an anabolic stimulus required to accelerate muscle recovery after eccentric damage, compared to placebo-treated mice. Whether 20E functions via the canonical protein synthesis pathway leading to recovery of skeletal muscle function needs to be investigated in future studies.

Skeletal muscle damage triggers a widespread series of events that can essentially be divided into two main stages: tissue degeneration and tissue regeneration. Tissue degeneration is necessary for removing damaged and dysfunctional structures, whereas tissue regeneration works to repair or rebuild muscle structures and regain function. Both stages rely heavily on mechanisms of inflammatory and myogenic pathways, not to mention many others. As discussed previously, inflammation is not responsible for the immediate decline of muscle function with eccentric contractions. However, inflammatory processes are essential for the successful regeneration of skeletal muscle and recovery of muscle function after injury (9). One of the hallmarks of histological muscle damage is the ordered infiltration of immune cells; first to respond are the neutrophils that appear in the first 24 h after injury, followed by M1-like macrophages around day 2, and finally M2-like macrophages by day 4 post-injury (8). In the current study we did not assess immune cells or inflammatory mechanisms, but phytoecdysteroids, including 20E, have been suggested to possess anti-inflammatory properties (27). While the anti-inflammatory effects of 20E directly on skeletal muscle are not known, previous reports suggest that the PI3K/Akt/mTORC1 pathway regulates immune (macrophage) cell function (63, 64). Theoretically, if 20E treatment is activating the PI3K/Akt/mTORC1 pathway during the 7-day recovery period, this could be potentially beneficial in managing the immune cell/inflammatory response to eccentric muscle damage and accelerating muscle recovery. However, we have no direct evidence that phytoecdysteroids influence inflammatory mechanisms during recovery from eccentric muscle damage, but this area certainly warrants further investigation.

Regarding our histological findings, it appears that markers of muscle damage are still apparent, albeit minimal, after 7 days of recovery from eccentric damage. It is interesting that despite a full recovery of muscle function (i.e., isometric torque) at 7 days in our 20E-treated groups, markers of muscle damage remain evident. This is not surprising as similar findings have been described previously wherein muscle function had recovered, but markers of muscle damage and regeneration, particularly centralized nuclei, are still visible weeks after eccentric contraction-induced muscle damage (35, 44). As described above, much of the inflammatory/immune response contributing to muscle regeneration has run its course by 7 days post-eccentric injury and what remains is tissue remodeling and growth processes. While the muscle tissue has been the main focus of this study, it is also important to recognize the importance of the association between the muscle fiber and connective tissue, or extracellular matrix (ECM), that surrounds the muscle fibers. Whether the ECM is damaged in response to eccentric contractions is still unclear (65). Generally speaking, aged skeletal muscle tissue contains more ECM than young muscle. One of the major complications with the age-related impairment in muscle regeneration after injury is not only the decreased myogenic potential, but also increased fibrogenesis (growth of connective tissue) (66). During regeneration of aged skeletal muscle, muscle tissue is replaced with connective tissue and, consequently muscle function and muscle quality decline. To our knowledge, no studies have investigated the effect of phytoecdysteroids on connective tissue or components of the ECM during muscle regeneration. If 20E is influencing connective tissue remodeling, it cannot be determined from our histological observations. Therefore, we cannot conclude whether 20E is providing any positive benefit to muscle damage markers or tissue remodeling in the 7-day recovery period after eccentric damage. However, future studies should investigate the potential role of 20E with regards to markers of muscle damage and tissue morphology with extended time points after eccentric muscle damage.

## Conclusion

In conclusion, eccentric contraction-induced damage results in significant declines in skeletal muscle function. Normal repair/regeneration of skeletal muscle tissue after damage relies on a series of highly-coordinated and time-dependent processes, including, but not limited to inflammatory, myogenic, and protein balance mechanisms. However, the regeneration process is impaired with aging. 20-hydroxyecdysone (20E) possesses anabolic, anti-inflammatory, and antioxidant properties. Here, for the first time, we demonstrate that daily treatment with 20E fully recovers skeletal muscle function in both adult and old mice within just 7 days after eccentric damage. The underlying mechanics by which 20E contributes to the accelerated recovery from muscle damage warrant further investigation. Taken together, it is reasonable to suggest that 20E has potential to be utilized as a supplementary intervention for muscle recovery after damage and in aging.

## Data Availability Statement

The original contributions presented in the study are included in the article/Supplementary Material, further inquiries can be directed to the corresponding author/s.

## Ethics Statement

The animal study was reviewed and approved by the Appalachian State University Institutional Animal Care and Use Committee.

## Author Contributions

KZ and RS were responsible for the conceptualization of the project, development of methodology, project administration, monitoring of data collection, statistical analysis, and writing of this manuscript. JG and CH were responsible for data collection, processing, and analysis and editing of this manuscript. All authors significantly contributed to the article and approved the submitted version.

## Funding

The authors would like to recognize the Appalachian State University Office of Student Research for providing partial funding for this project.

## Conflict of Interest

The authors declare that the research was conducted in the absence of any commercial or financial relationships that could be construed as a potential conflict of interest.

## Publisher's Note

All claims expressed in this article are solely those of the authors and do not necessarily represent those of their affiliated organizations, or those of the publisher, the editors and the reviewers. Any product that may be evaluated in this article, or claim that may be made by its manufacturer, is not guaranteed or endorsed by the publisher.
