# Supplementary material for: Phytoecdysteroids Accelerate Recovery of Skeletal Muscle Function Following in vivo Eccentric Contraction-Induced Injury in Adult and Old Mice
Source: Front Rehabil Sci. 2021 Nov 8;2:757789. doi: 10.3389/fresc.2021.757789 (PMC9397830; doi:10.3389/fresc.2021.757789)
Supplement: Supplementary file 1 [file Data_Sheet_1.docx]

Supplementary Material

**SUPPLEMENTARY TABLE 1 |** Animal and muscle characteristics of sham-treated groups.

Values represent mean ± SEM. PLA, placebo; 20E, 20-hydroxyecdysone; TA, tibialis anterior; EDL, extensor digitorum longus. $ = significantly different than Initial (p<0.05); # = significantly different than Adult (p<0.001).

Supplementary Figure Legends

**Supplementary Figure 1.** Isometric Torque-Frequency Relationships of the anterior crural muscles in sham-treated Adult and Old mice at the Initial (A and D, respectively), Post (B and E, respectively), and 7-day recovery (C and F, respectively) time points. Sham-treated mice performed all muscle function tests and daily treatment regimens, but were not subjected to the eccentric contraction-induced muscle damage protocol. Analysis revealed that there were no differences in isometric torque-frequency relationships in either age group, regardless of time point or treatment condition (p>0.05 for all). Values represent mean ± SEM. PLA, placebo; 20E, 20-hydroxyecdysone; Initial, initial muscle function test; Post; second muscle function test at the time point equivalent to post-injury in eccentric damage groups; 7-day, third muscle function test at the same time point as 7-day recovery post-injury in eccentric damage groups.

**Supplementary Figure 2.** Representative images for H&E-stained tibialis anterior (TA) muscle sections from sham-treated (Sham) and eccentric damaged (Injured) groups after the 7-day recovery period with Muscle Damage Scores indicated for each image. H&E staining procedures provide visualization of the nuclei (dark blue) and cytosol (pink). Injured TA muscle sections appear to have more markers of muscle damage (e.g., edema, overt fiber damage, presence of infiltrating inflammatory cells, and centrally-located myonuclei), regardless of treatment, compared to Sham muscle sections. Muscle Damage Scores are based on a scale of 0-3, with 0 = no apparent muscle damage; 1 = minimal muscle damage; 2 = moderate muscle damage; and 3 = severe muscle damage. Muscle damage scores appear to be higher in the PLA-treated groups, compared to 20E-treated groups, but the old PLA-treated mice appear to have the greatest muscle damage score at 7 days post-injury, compared to any other group. PLA, placebo; 20E, 20-hydroxyecdysone. Scale bar = 100 µm.

Supplementary Figure 1.

Supplementary Figure 2.


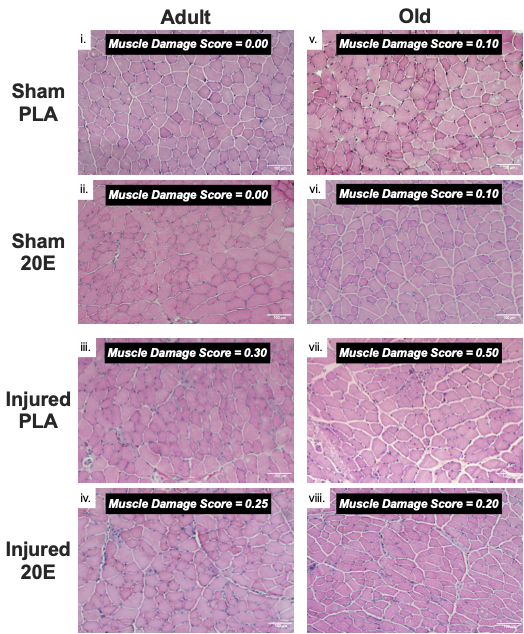


**
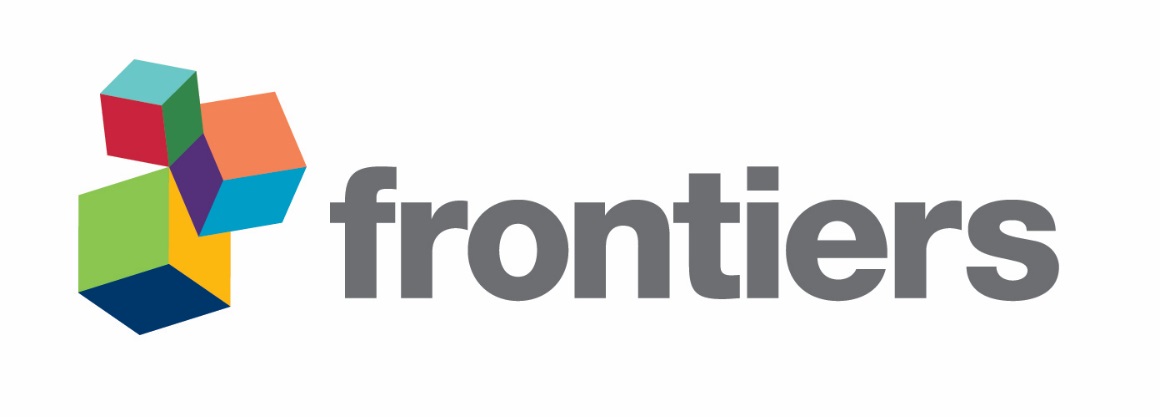
**
